# A comparison of genomic laboratory reports and observations that may enhance their clinical utility for providers and patients

**DOI:** 10.1002/mgg3.551

**Published:** 2019-05-21

**Authors:** Kyle Walter Davis, Lori Hamby Erby, Katie Fiallos, Megan Martin, Edward Robert Wassman

**Affiliations:** ^1^ Lineagen, Inc. Salt Lake City Utah; ^2^ National Human Genome Research Institute, National Institutes of Health Bethesda Maryland; ^3^ Sidney Kimmel Cancer Center, Johns Hopkins School of Medicine Baltimore Maryland

**Keywords:** genetic testing, genomics, health communication, health literacy, laboratory reports, patient communication, physician–patient relations, provider communication, shared decision making

## Abstract

**Purpose:**

To assess clinical chromosomal microarray (CMA) genomic testing reports for the following: (a) usage of reporting elements consistent with 2011 ACMG guidelines and other elements identified in the primary literature, (b) information quality, and (c) readability.

**Methods:**

We retrospectively analyzed genomic testing reports from 2011 to 2016 provided to, or by our laboratory to aid in clinical detection and interpretation of copy number variants. Analysis was restricted to the following sections: interpretation, recommendations, limitations, and citations. Analysis included descriptive characteristics, reporting elements, reading difficulty using the Simple Measure of Gobbledygook (SMOG), and quality ratings using a subset of questions adapted from the DISCERN‐Genetics questionnaire.

**Results:**

The analysis included 44 unique reports from 26 laboratories comprising four groups: specialty laboratories (SL; *N* = 9), reference laboratories (RL; *N* = 12), hospital laboratories (HL; *N* = 10), and university‐based laboratories (UL; *N* = 13). There were 23 abnormal/pathogenic reports and 21 of uncertain/unknown significance. Nine laboratories did not include one or more pieces of information based on ACMG guidelines; only one of ten laboratories reported condition‐specific management/treatment information when available and relevant. Average quality ratings and readability scores were not significantly different between laboratory types or result classification.

**Conclusions:**

Reporting practices for most report elements varied widely; however, readability and quality did not differ significantly between laboratory types. Management and treatment information, even for well‐known conditions, are rarely included. Effectively communicating test results may be improved if certain reporting elements are incorporated. Recommendations to improve laboratory reports are provided.

## 1 INTRODUCTION

As knowledge of genetic contribution to disease increases, so will the use of genomic testing, including chromosomal microarray analysis (CMA) and exome sequencing. Multiple professional medical organizations and pediatric experts recognize the utility of genomic tests, such as microarray testing, and recommend its use in practice guidelines and other publications (Manning & Hudgins, 2010; Miller et al., 2010; Satya‐Murti, Cohen, & Michelson, 2013; Volkmar et al., 2014). Although genetics clinics will likely remain the primary medical “home” for people with rare genetic conditions, diagnosis and/or initial treatment and management are increasingly likely to occur within the context of other clinical settings. By the year 2020, genomic medicine is anticipated to be regularly used to improve health care (Green & Guyer, 2011). Indeed, diagnostic clinical testing is already moving from genetic specialists, such as geneticists, to non‐genetic specialists, such as neurologists, and generalists, such as pediatricians. Accordingly, medical providers, regardless of specialty, will increasingly use genomic testing and encounter results that range from well‐known conditions to those associated with rare conditions, and chromosomal rearrangements that are unique to an individual.

Communicating these complex results effectively and clearly is recognized as a challenge for both providers and patients/families (Haga et al., 2014; Vassy et al., 2015). As such, genetic testing reporting practices have been the subject of the previous study. This small but growing literature has focused on several elements of reporting practices, including the optimal layout/formatting for a report and specific types of information to include (reporting elements).

To make communicating genomic results more uniform, the American College of Medical Genetics (ACMG) published standards and guidelines for reporting copy number variants (CNVs) in genomic laboratory reports in 2011 (Kearney, Thorland, Brown, Quintero‐Rivera, & South, 2011). These standards included eight content elements. In addition, several studies have examined providers’ reporting preferences for other elements that may be desirable (Haga et al., 2014; Scheuner, Edelen, Hilborne, & Lubin, 2013; Scheuner, Hilborne, Brown, & Lubin, 2012; Stuckey et al., 2015; Williams et al., 2016). Non‐ACMG elements identified in the literature include the following: Model formatting of the report, information presented in a table, the reason the test was ordered (e.g., the diagonals code), references to practice guidelines (i.e., treatment or management information), interactive hyperlinks to relevant information, patient resources, secondary findings, potential “next steps,” and a “patient‐facing” report written for the patient with minimal technical or medical jargon and using simpler language overall (Haga et al., 2014; Williams et al., 2016). Collectively, these studies yield at least 15 total elements a laboratory could include in a report (Table 1).

**Table 1:** List of content and style elements in clinical genetics reports

ACMG‐recommended guideline elements	Non‐ACMG reporting elements
*Cytogenetic location*: The location on the chromosome of the genetic alteration (e.g., 1q21.1)	*Model‐style formatting*: A dedicated genetics report template with headings and sections (see Supporting information, Figure S1)
*CNV size*: The total size in bases of the genetic alteration (e.g., 139 kilobases)	*Table(s) used for information*: A table summarizing key information about the genomic alteration (e.g., gene content, cytogenic location, classification)
*CNV linear coordinates*: The coordinates that correspond to the genetic alteration (e.g., chr1:142,600,001‐155,000,000)	*Testing indication(s)/diagnosis code(s)*: The symptom(s) or diagnosis code(s) relevant to testing (e.g., “developmental delays” or R62.0)
*Gene dosage*: The number of copies of a gene or region (e.g., “copy number gain” or “copy number loss”; or written in ISCN nomenclature)	*Treatments/managements referenced*: A statement in the regarding known treatment or management information (e.g., Williams syndrome health supervision guidelines)
*Gene content*: A list of the gene(s) located within the genetic alteration	*Clinical resource hyperlinks*: Links to additional clinical information (e.g., a link to GeneReviews)
*Classification statement*: A statement of significance about the genetic alteration (e.g., “pathogenic”)	*Patient resources hyperlinks*: Links to a specific condition or support and/or educational group for patients or families for (e.g., Bright Pink)
*Literature cited*: References cited that support the interpretation and classification	*Patient‐facing report*: A lay interpretation of some or all of the clinical report
*Follow‐up recommendations*: Additional actions for the provider or family based on the genetic alteration (e.g., parental studies to determine inheritance)	

Effective communication of a genomic finding is essential to fully realize the many benefits of a genetic test result for the patient and his/her family. Concerns have been noted about challenges providers face with interpreting reports (Dhar et al., 2011; Haga et al., 2014; Julian‐Reynier, Eisinger, Moatti, & Sobol, 2000; Munson & Leuthner, 2007; Pal et al., 2014; Vassy et al., 2015). In particular, evidence has suggested that nongenetics specialists may need assistance with understanding the implications of genetic test results for patient management (Dhar et al., 2011; Julian‐Reynier et al., 2000; Pal et al., 2014).

Although there is no definition of “effective communication” in a laboratory report, several factors are likely to influence communication of technical writing. These may include writing text at an appropriate level for nongenetics providers, as communication and comprehension of a text depend in part on readability, regardless of the literacy level of the intended audience (Foe & Larson, 2016; Friedman & Hoffman‐Goetz, 2007; Kušec et al., 2006). Other aspects of “effective communication” likely include clearly formatting important information, incorporating quality information including preferred reporting details (i.e., “non‐ACMG elements”), and providing follow‐up recommendations (Haga et al., 2014; Scheuner et al., 2012; Williams et al., 2016).

Indeed, providers more often support the “Model” format (Scheuner et al., 2012) for reports which clearly organizes patient, clinical, and other information into discrete categories and uses headings and subheadings and stylistic differences like bold font to guide the reader through the report. This format is associated with higher provider ratings of satisfaction, ease of use, and overall effectiveness of conveying information (Scheuner et al., 2013). Inclusion of condition guidelines or recommendations in laboratory reports can be viewed as “too prescriptive” (Scheuner et al., 2012), but a newer study has found that providers do approve of referencing condition‐specific guidelines while using terminology to describe these as “considerations” (Williams et al., 2016). When this language is used, providers have strongly supported test reports that include references such as practice guidelines. Considering there are currently over 5,900 phenotypes for which a molecular basis is defined in the Online Mendelian Inheritance in Man (OMIM) database and that at least 3% of adults are ostensibly affected with an identifiable genetic condition (Johnston et al., 2015), clinicians cannot be expected to keep abreast of this highly dynamic knowledge base, especially as this number is expected to rise given more refined understanding of genetic contribution to disease.

Despite recent analyses of how providers and patients prefer to have information presented in genomic laboratory results (Scheuner et al., 2013, 2012), to our knowledge, there has not yet been a systematic exploration or analysis of the content and readability of genetic testing reports. An analysis of reporting practices can identify strengths and shortcomings that are inherent to communicating complex genomic results, which may lead to improving communication of genetic findings, better equipping providers with clearer and more effective reports, and ultimately improving patient care. As genomic knowledge and testing matures, providing a report that is high‐quality, readable, easy to use, informative and actionable genomic will be paramount.

The goal of this study was to describe the current landscape of genomic reports from several groups of laboratories. To do this, we analyzed genomic testing reports for CMA from our laboratory or CMA or array comparative genomic hybridization (aCGH) reports from other laboratories submitted to us by providers or patients. This is a descriptive analysis that aimed to provide insight into the use of recommended reporting practices that could ultimately benefit the provider and the patient.

## 2 MATERIALS AND METHODS

### 2.1 Report selection

The dataset represents a retrospective sample of unsolicited genetic testing reports submitted to our laboratory by families, patients, or providers to aid with genetic testing interpretation in the course of routine patient care. The publication date of the ACMG reporting guidelines, 2011, was used as a cutoff for inclusion. Thus, the inclusion criteria were as follows: (a) reports for a CMA or aCGH testing; (b) reports from 2011 or after (after publication of the ACMG reporting guidelines); and (c) test results for an “abnormal” or pathogenic alteration or a result of “uncertain significance” (variant of uncertain/unknown significance; VOUS). We attempted to include an equal number of pathogenic and VOUS results. Additionally, no single laboratory represented more than 25% of the total sample within its specific analysis category. Two reports are included from our own laboratory, one VOUS report and one pathogenic report for a pediatric condition commonly diagnosed by CMA.

### 2.2 Laboratory and classification categorization

Diagnostic laboratories were categorized as specialty, reference, academic/university‐based, or hospital‐based. These categories were created for this analysis primarily based on scope of testing and setting as no such pre‐existing categorization was available. Specialty laboratories were defined as laboratories that specialize in one or more types of genetic testing and do not perform other categories of testing. Reference laboratories were defined as any laboratory that does not specialize in a specific type of test and is not located at a hospital or university. Academic/university‐based laboratories were classified as any laboratory that is affiliated with an academic institution. Hospital‐based laboratories were classified as any laboratory that performs testing for a hospital or hospital system and is not affiliated with a university. No laboratory appeared in more than one category.

Individual laboratory reports were categorized either as pathogenic/abnormal or as variants of uncertain/unknown significance. If a report contained a pathogenic finding and one or more uncertain finding(s), then it was classified as abnormal/pathogenic. In two instances, there was no indication on the report of classification, but the interpretation used wording that implied that the finding was of an uncertain clinical nature and thus these were categorized as unknown/uncertain findings.

### 2.3 Descriptive and readability analyses

Text was extracted from the interpretation, recommendations, citations, and limitations sections of each report. Explanations of testing methodology, testing uses, and descriptions of the test product were excluded from analyses. Although important, these sections were deemed least likely to directly impact and guide clinical care. Of this dataset, we had access to the full report for 39 reports while five were partial reports. All items were assessed when possible, and when an item was unable to be assessed, it was not included in analyses. Statistical analyses were performed in Stata version 12.

We documented descriptive details such as year reported, format style, total length in pages, total word count, characteristics of citations, and patient resources. We counted the total number of citations in a report (continuous variable) as well as whether a report cited the primary literature or an online database, such as OMIM (categorical variables).

We also reviewed each report for the eight elements specifically described in the 2011 ACMG guidelines and the seven elements from our nonsystematic literature review (Table 1). The patient‐facing report was defined as a summary of the result intended for the patient or a dedicated report discussing the result for the patient, as discussed in Haga et al. (2014). Each report from each laboratory was reviewed for all elements, and each element was recorded as a categorical variable (Yes vs. No). A “yes” was recorded if any report by the laboratory included the element. Thus, if a “yes” was recorded, the specific element was included in at least one report from the laboratory, and when a “no” was recorded, all reports from that laboratory lacked the element entirely. These data are presented at a laboratory level (as opposed to an individual report level) in an effort to account for changes in reporting characteristics within a laboratory and to try to assess the general reporting practices. Of note, the treatment and management information element was not assessed for uncertain/unknown findings, findings where treatment information was published after the report was issued, or findings/conditions where there is no published treatment or management information.

To measure reading level, all de‐identified text from the previously mentioned sections was extracted for analysis using the Simple Measure of Gobbledygook (SMOG). The SMOG score estimates the number of years of education a person needs to understand a given passage, such that a score of 12 would require an educational level of a high school senior and a 16 the educational level of a college senior. It is most often used in evaluating written healthcare information, is recommended by several organizations, and correlates well with understanding about 90%–100% of a text for a given grade level (Luk & Aslani, 2011).

In accordance with readability analysis using the SMOG formula, we replaced acronyms (e.g., “CMA”) with the full‐length term (e.g., “chromosomal microarray analysis”). The only instance where this was not performed was for gene names. To address this issue, we replaced the gene's acronym with the word “genetic” as a multisyllabic word and a general proxy. Although this does somewhat artificially lower the SMOG score, this method may be closer to the way in which providers and patients read a gene name. (e.g., this can be illustrated using the *ABCD1* (OMIM ID: 300371), which is mostly likely read as “a‐bee‐cee‐dee‐one” and not as the ATP‐binding cassette, subfamily D, member 1 gene.) Transformed text was entered into an online SMOG calculator tool to determine the final score for each report. The SMOG tool is located at: http://wordscount.info/wc/jsp/clear/analyze_smog.jsp.

Lastly, we grouped the same findings together to contrast variability in reporting practices. We identified three results that were reported three times each in this series: 22q11.2 deletion syndrome, Wolf–Hirschhorn syndrome, and 15q11.2 BP1‐BP2 deletions. Statistical testing was not performed on this subset of reports, and reporting elements were not ascertained (as these elements were reported at the laboratory level and not the report level).

### 2.4 Quality analyses

To assess report quality, we used the DISCERN‐Genetics tool, which measures the quality of information for genetic testing (Shepperd et al., 2006). This tool was developed by a group of healthcare and genetics professionals to determine the quality of information about genetic testing presented to the public (e.g., a brochure for a genetic test for cystic fibrosis). Although the tool is designed to help determine information quality about a genetic test, several questions discuss information often included in a genetic testing report. As such, we used specific questions from this tool to as a way to gauge overall quality of a report by using five questions, corresponding to questions 3–5 and 15–16. These were deemed most appropriate to adapt for quality assessment, as other questions are intended to assess other aspects of information quality for genetic testing (e.g., a document's aims/goals or identifying bias in a publication). The five items chosen to assess report quality were as follows: (a) an explanation of the background and effects of a given result, (b) descriptions of treatments and management, (c) an explanation of risk for the person receiving results as to whether they have a condition or the risk that they will develop symptoms of a condition, (d) providing additional support resources, and (e) clarity of the sources used for the report (i.e., citations).

The first three items (background, risk, and treatment and management) are classified in the DISCERN‐Genetics tool as “Background information,” while the last two items (patient support and sources) are classified as “Information Reliability.” Thus, three overall measures are reported for the quality assessment including the average total quality score, average Background Information score, and average Information Reliability score. Rating schema was adapted for this study by one rater (KD), but included original rating guidelines when possible. For example, the first question (background and effects of a result) requires different scoring definitions for pathogenic findings versus uncertain or unknown findings. There is often specific background information about a condition, whereas an uncertain finding will have much more limited information available. The rating schema differed between pathogenic and unknown reports only for the first question, regarding background information on the condition.

Similarly, we did not assess the two questions regarding (a) risk and (b) treatments and management for reports classified as unknown/uncertain/indeterminate, as there are no treatments for unknown findings and risk of an individual developing a condition is likewise unknown. Similarly, quality ratings for treatments and management were not assessed for reports of conditions which do not have guidelines or recommendations or for which guidelines or recommendations did not exist at the time the report was completed.

Each of the five DISCERN questions was graded on a 5‐point scale, with a score of 5 being the highest quality and a score of 1 the lowest indicating a complete lack of the quality measure. Thus, the lowest possible raw score for an individual report was either a 3 (for “unknown” findings) or a 5 (for pathogenic findings) and the highest possible score was a 15 or a 25, respectively. To allow for comparison between results, these scores were then averaged, such that a “perfect” report for an unknown finding would have the same average score as a “perfect” report for a pathogenic finding (15 total points divided by three questions yielding an average 5.0 vs. 25 total points divided by five questions yielding the same 5.0 average).

Two raters (KD and KF) independently provided ratings for all questions of all reports. Both raters are certified genetic counselors, trained at the same genetic counseling program, and have previously used the DISCERN‐Genetics tool. Thus, they were familiar with the questions and rating system. Due to logistical difficulties, raters were not blinded to the source of the reports (i.e., the laboratory name). Inter‐rater reliability was calculated for quality ratings using two Kappa statistics. A previous weighted Kappa statistic for this tool is reported as 0.60–0.62 (Shepperd et al., 2006).

### 2.5 Statistical analyses

Bivariate relationships between key report variables were explored using Pearson's correlation analyses. ANOVA was used to analyze for differences in report characteristic and reporting elements between reports from different laboratory types as well as between reports classified as pathogenic and uncertain significance. Bonferroni correction was used when comparing differences between laboratories. Chi‐squared analyses were used to analyze between‐group differences in categorical variables, such as specific reporting elements. Lastly, multiple linear regression analyses were performed to determine which variables were significantly associated with quality and readability. We used a reverse elimination method to arrive at the final model by first using all variables in the model, then removing all variables with *p* > 0.25. The remaining variables were used to build the final model. All findings were considered significant at *p* ≤ 0.05.

For DISCERN‐Genetics quality scores, all scores were standardized by obtaining the average score of the items that could be scored. Thus, all average scores had a possible minimum rating of 1.0 and a possible maximum rating of 5.0. Inter‐rater reliability between KD and KF was determined using two weightings for the Cohen's Kappa statistic. The first was a nonweighted (“standard”) version that is preprogramed with Stata 12. The second, weighted version was user‐defined. The weighted Kappa statistic was designed to be more stringent by the following: (a) counting only perfect agreement for the highest and lowest ratings (5 or 1), meaning there was no partial scoring credit if there was not perfect agreement between the two raters and (b) counting only perfect ratings or ratings that differed by one point for scores between 2 and 4. Thus, there was no partial scoring credit if ratings differed by ≥2 points for any given question's rating.

### 2.6 Ethical compliance

Data collection and analysis for this study have been approved by Western IRB (study number 20162032).

## 3 RESULTS

A total of 44 reports representing 26 unique laboratories were analyzed. These were split across four broad laboratory categories: specialty laboratories (SL), reference laboratories (RL), hospital‐based laboratories (HL), and university‐based laboratories (UL). Within these groups, the total number of reports and percentage of the total were SL: *N* = 9; 20%, RL: *N* = 12; 27%, HL: *N* = 10; 23%, and UL: *N* = 13; 30%. Six different laboratories were included in the SL group, four in the RL group, eight in the HL group, and eight in the UL group.

The report characteristics are described in Table 2. SLs used the Model format for all reports, while ULs, HLs, and RLs used it less often (69%, 30%, and 8%, respectively). Word count ranged from an average of 365.8 (ULs) to 232.3 (HLs). The shortest report across all laboratories was 67 words, while the longest was 659 words. Page length of reports ranged from 1 page (SLs) to 6 pages (ULs), though the average for all laboratories was between 2.2 (HLs) and 3.0 (SLs). Similarly, total citations varied considerably from a low of zero (recorded in at least one report for all laboratory types) to 10 (SLs), with average total citations between 2.3 (ULs) and 3.9 (SLs).

**Table 2:** Descriptive statistics of all genomic reports by laboratory and classification

	Laboratory type				Classification		All
	SL	RL	HL	UL	Pathogenic	VOUS	
Total reports	9	12	10	13	23	21	44
Pathogenic classification	3	7	6	6	N/A	N/A	N/A
VOUS classification	6	5	4	7	N/A	N/A	N/A
Years included	2011–2016	2011–2016	2011–2015	2012–2016	2011–2016	2011–2015	2011–2016
Model format							
Total	9	1	3	9	N/A	N/A	N/A
Percentage	100%	8%	30%	69%	N/A	N/A	N/A
Word count							
Range	132–639	67–551	103–403	107–659	67–659	107–639	67–659
Average	358.9	311.2	232.3	365.8	317.8	319.1	318.5
Median	161.2	141.9	229	324	287.0	292.0	298.5
Document length (pages)							
Range	1–4	1–5	1–4	1–6	1–6	1–5	1–6
Average	3.0	2.8	2.2	2.3	2.6	2.6	2.5
Median	3.0	3.0	2.0	2.0	2.0	3.0	2.5
Total citations							
Range	0–10	0–7	0–7	0–6	0–10	0–10	0–10
Average	3.9	3.4	2.4	2.3	3.0	2.9	3.0
Median	2.0	3.5	2.5	2.0	3.0	2.0	2.0

Note

SL: specialty laboratory; RL: reference laboratory; HL: hospital‐based laboratory; UL: university‐based laboratory; N/A: not applicable; VOUS: variant of uncertain/unknown significance.

There were few differences between characteristics for reports of different classifications (pathogenic/abnormal vs. unknown/uncertain significance). None of the differences were statistically significant, though VOUS results tended to be longer in total pages (median of 3.0 vs. 2.0) and word count (median of 292 vs. 287), though they also tended to use fewer citations (median 2.0 vs. 3.0).

### 3.1 Analyses of descriptive characteristics of reports

There were statistically significant differences between laboratory types for several report characteristics. Although SL reports were longer in terms of word count and document length, and used more citations, these differences were not statistically significant between laboratory types (Table 2). There were statistically significant differences regarding the use of Model formatting, with SL reports being more often formatted in this style than RL and HL (*X* ^2^ = 17.3, *p* < 0.0001 and *X* ^2^ = 9.9, *p* = 0.002, respectively) and UL reports more likely to use Model formatting than RL (*X* ^2^ = 9.6, *p* = 0.002).

There were no significant differences between reports for pathogenic variants as compared to reports for variants with unknown significance with regard to average report length, word count, or total citations.

### 3.2 Reporting elements

**Figure 1:**
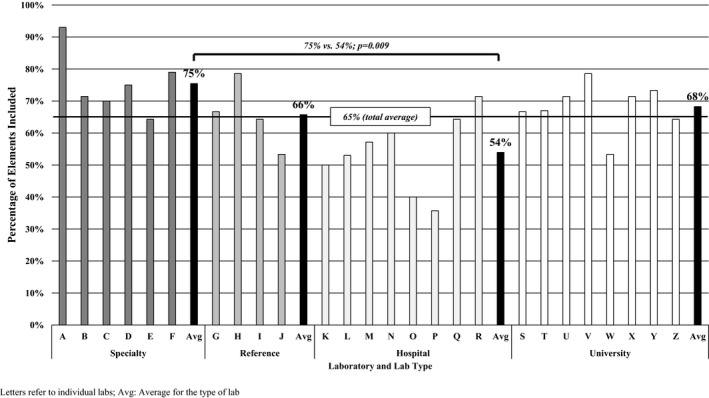
The total percentage of laboratory elements included by each individual laboratory and averaged across laboratory type

The proportion of total reporting elements included in the reports ranged from 36% (Lab P) to 93% (Lab A). By using two‐sided *t* tests, we found that ACMG reporting elements were more often included in reports than non‐ACMG elements (93% vs. 34%, *p < *0.0001). This remained true when analyzing ACMG versus non‐ACMG elements within each laboratory types (SLs: 98% vs. 52%, *p = *0.001; RLs: 97% vs. 33%, *p = *0.05; HLs: 84% vs. 18%, *p < *0.0001; ULs: 96% vs. 38%, *p < *0.0001). Between laboratories, SLs included more total elements than HLs (74% vs. 54%, *p = *0.009) and more non‐ACMG elements (52% vs. 18%, *p = *0.022). A summary of all laboratories and their inclusion of specific reporting elements can be found in Table 3 and Figure 1.

**Table 3:** Specific reporting elements by laboratory

Laboratory		ACMG‐recommended guideline elements								Non‐ACMG report elements							Totals		
Type	Laboratory	1	2	3	4	5	6	7	8	9	10	11	12	13	14	15	ACMG (%)	Others (%)	Total (%)
SL	Lab A	Y	Y	Y	Y	Y	Y	Y	Y	Y	Y	Y	Y	Y	Y	N	100	86	93
	Lab B	Y	Y	Y	Y	Y	Y	Y	Y	Y	Y	N	‐	N	N	N	100	33	71
	Lab C	Y	Y	Y	‐	‐	‐	Y	Y	Y	N	‐	‐	Y	N	N	100	40	70
	Lab D	Y	Y	Y	Y	Y	Y	Y	‐	Y	N	Y	‐	‐	N	N	100	40	75
	Lab E	Y	Y	Y	Y	Y	Y	N	Y	Y	N	Y	‐	N	N	N	88	33	64
	Lab F	Y	Y	Y	Y	Y	Y	Y	Y	Y	N	Y	‐	Y	N	N	100	50	79
RL	Lab G	Y	Y	Y	Y	Y	Y	Y	Y	N	N	Y	N	Y	N	N	100	29	67
	Lab H	Y	Y	Y	Y	N	Y	Y	Y	Y	N	Y	‐	Y	Y	N	88	67	79
	Lab I	Y	Y	Y	Y	Y	Y	Y	Y	N	N	Y	‐	N	N	N	100	17	64
	Lab J	Y	Y	Y	Y	Y	Y	Y	Y	N	N	N	N	N	N	N	100	0	53
HL	Lab K	Y	Y	Y	Y	N	N	Y	Y	N	N	Y	‐	N	N	N	75	17	50
	Lab L	Y	Y	Y	Y	Y	Y	Y	N	N	N	Y	N	N	N	N	88	14	53
	Lab M	Y	Y	Y	Y	Y	Y	Y	N	Y	N	N	‐	N	N	N	88	17	57
	Lab *N*	Y	Y	Y	Y	Y	Y	Y	N	N	N	Y	N	Y	N	N	88	29	60
	Lab O	Y	Y	Y	Y	N	Y	N	Y	N	N	N	N	N	N	N	75	0	40
	Lab P	Y	Y	Y	Y	N	Y	N	N	N	N	N	‐	N	N	N	63	0	36
	Lab Q	Y	Y	Y	Y	Y	Y	Y	Y	Y	N	N	‐	N	N	N	100	17	64
	Lab R	Y	Y	Y	Y	Y	Y	Y	Y	Y	N	Y	‐	N	N	N	100	33	71
UL	Lab S	Y	Y	Y	Y	Y	Y	Y	Y	Y	N	Y	N	N	N	N	100	29	67
	Lab T	Y	Y	Y	Y	Y	Y	Y	Y	Y	N	Y	N	N	N	N	100	29	67
	Lab U	Y	Y	Y	Y	Y	Y	Y	Y	Y	N	Y	‐	N	N	N	100	33	71
	Lab V	Y	Y	Y	Y	Y	Y	Y	Y	Y	Y	Y	‐	N	N	N	100	50	79
	Lab W	Y	Y	Y	Y	Y	Y	N	N	Y	N	Y	N	N	N	N	75	29	53
	Lab X	Y	Y	Y	Y	Y	Y	Y	Y	Y	N	Y	‐	N	N	N	100	33	71
	Lab Y	Y	Y	Y	Y	Y	Y	Y	Y	Y	Y	Y	N	N	N	N	100	43	73
	Lab Z	Y	Y	Y	Y	Y	Y	Y	Y	N	N	Y	‐	N	N	N	100	17	64
Total		100%	100%	100%	100%	84%	96%	85%	80%	65%	15%	76%	10%	24%	8%	0%			

Note

1: cytogenic location; 2: CNV size; 3: CNV linear coordinates; 4: gene dosage (e.g., deletion); 5: gene content; 6: classification statement (e.g., “pathogenic”); 7: literature citations; 8: follow‐up recommendations; 9: Model format; 10: information presented in table format; 11: Indication/Diagnosis codes included; 12: treatments/managements referenced for specific conditions; 13: clinical resource hyperlinks; 14: patient resource hyperlinks; 15: patient‐facing content report.

Y: Yes/included; N: No/omitted; dash: could not be assessed due to incomplete records.

A majority of laboratories (17 of 26; 65%) included all ACMG reporting elements. However, laboratories ranged from including five of the eight elements (63%) to all eight. Using one‐way ANOVA analyses, no statistical differences were found between laboratory type. When analyzing reports by specific elements, only four elements were included by all laboratories. These four elements were cytogenetic location, CNV size, CNV linear coordinates, and gene dosage (i.e., deletion or duplication). The elements that were not routinely addressed on at least one laboratory report by each laboratory, and the proportion of laboratories that included them, were as follows: variant classification statement (96% included), literature citations (85%), gene content (84%), and follow‐up recommendations (80%). In general, HLs were the least likely to include all ACMG reporting elements (only two of eight laboratories), while a majority of laboratories in all other laboratory types reported at or near 100%.

Of the seven non‐ACMG elements, only four laboratories included at least 50% (when these elements were able to be assessed). One laboratory included six of seven total elements, while one other laboratory included four of six possible elements (the seventh element, treatment and/or management guidelines, could not be assessed for one of the reports from this laboratory). Overall, the inclusion of diagnosis codes or testing indications and using the Model formatting were much more commonly observed than the other non‐ACMG elements, with 76% and 65% of laboratories using these elements, respectively. Three elements were often omitted from a report: hyperlinks to clinical resources (24%), a table to organize reported information (15%), references to treatment or management resources (9%), hyperlinks to patient resources (8%), and patient‐ or family‐facing report (0%). SLs included statistically significantly more of these non‐ACMG elements than HLs (52% vs. 18%, *p = *0.02).

### 3.3 Readability

Average SMOG scores were similar across all laboratory types: 17.1 (SL), 17.2 (UL), 17.4 (HL), and 17.8 (RL). The range of scores was largest for HLs and SLs at 5.5 grade levels and smallest for ULs at 4.9 grade levels. There were no statistically significant differences between SMOG scores across laboratory type. There were statistically significant differences based on result classification, with pathogenic reports being almost one full grade level more difficult to read based on single linear regression analysis (*β* = 0.99, *p* = 0.03). However, this difference was no longer statistically significant when adjusting for other variables in a multiple linear regression analysis (*β* = 0.85, *p* = 0.07). Other variables significantly associated with readability in the final multiple linear regression model were word count (*β* = 0.004, *p* = 0.03) and number of primary literature citations (*β* = −1.79, *p* = 0.02). The final model predicted ~14% of the variance in report readability (adjusted *r^2^*).

### 3.4 Quality

#### 3.4.1 Inter‐rater reliability

When using the standard Stata weighting calculation to compare the quality ratings for KD and KF, all five items showed statistically significant rating agreement between raters (see Supporting information Table S1). Depending on the item, percent agreement ranged from 28.0% to 92.3% and Cohen's Kappa ranged from 0.14 to 0.64 (*p* < 0.05 for all). To generate an overall Kappa statistic, we averaged all five items to yield a rating of 0.46, which is considered “moderate” agreement (Hallgren, 2012). When using the unique weighting calculation, four of five items showed statistically significant rating agreement. Of the ratings that reached statistical significance, the percent agreement ranged from 68.2% to 92.3% and Cohen's Kappa ranged from 0.49 to 0.65 (*p* < 0.001 for all), with an average Kappa of 0.61, which is considered “substantial.” The one area in which the two raters’ ratings did not agree significantly was for risk (percent agreement: 34.4%; Cohen's Kappa: 0.09; *p* = 0.17).

To analyze for potential bias between raters, two‐way *t* tests were performed on overall quality ratings by laboratory type. When comparing the ratings by KD and KF, no statistical differences were observed between ratings for SLs, RLs, and ULs (all *p ≥ *0.2). Total quality ratings differed between only HLs (KD: 2.39 vs. KF: 2.18, *p* = 0.02). Thus, our total quality ratings were similar for most laboratory types.

#### 3.4.2 Quality scores

Total quality scores ranged widely for individual reports for all laboratory types for both raters, though average total quality ratings were similar across laboratory types (Table 4). At least one report from each laboratory type rated by each rater received a score below 2, meaning the report lacked all or almost all assessed quality areas, while other individual reports were rated at a 3.5–5.0 by each rater. The combined, average total quality scores were from 2.29 to 3.12; combined average Background Information ranged from 2.17 to 3.36; and combined Information Reliability ranged from 2.39 to 2.92.

**Table 4:** Detailed quality ratings by rater

	KD ratings							KF ratings				Combined average ratings			
	SL	RL		HL		UL		SL	RL	HL	UL	SL	RL	HL	UL
Total quality															
Mean (*SD*)	3.3 (1.53)	2.9 (1.07)		2.4 (0.92)		2.5 (0.98)		2.9 (1.59)	2.9 (1.08)	2.2 (0.96)	2.5 (1.05)	3.1	2.9	2.3	2.5
Median	3.3	2.8		2.2		2.7		3.3	3.0	1.8	2.6	‐	‐	‐	‐
Range	1–5	1–5		1.2–3.7		1–4.7		1–5	1.3–4.7	1–3.7	1–4	‐	‐	‐	‐
Background Information quality															
Mean (*SD*)	3.6 (1.65)		3.4 (1.09)		2.3 (0.85)		3.0 (1.56)	3.1 (1.54)	3.2 (0.67)	2.0 (0.91)	2.8 (1.34)	3.4	3.3	2.2	2.9
Median	4.0		3.5		2.3		3.0	3.0	3.0	1.8	3.0	‐	‐	‐	‐
Range	1–5		1–5		1.3–3.5		1–5	1–5	2–4	1–3.3	1–5	‐	‐	‐	‐
Information Reliability quality															
Mean (*SD*)	3.0 (1.58)		2.42 (1.41)		2.6 (1.26)		2.2 (1.17)	2.8 (1.73)	2.6 (1.45)	2.5 (1.33)	2.5 (1.36)	2.9	2.6	2.6	2.4
Median	3.5		2.3		3.0		2.0	3.0	3.0	3.0	3.5	‐	‐	‐	‐
Range	1–5		1–5		1–4		1–4.5	1–5	1–5	1–4	1–4	‐	‐	‐	‐

Note

SL: specialty laboratory; RL: reference laboratory; HL: hospital laboratory; UL: university‐based laboratory.

Specialty laboratories consistently scored higher than other laboratory types for all three quality categories, while HLs scored lowest for total quality and Background Information and ULs score lowest for Information Reliability. When averaging the quality scores between the two raters, the highest single score in any category was for Background Information provided by SLs (3.36), while the lowest single score in any category was for Background Information provided by HLs. However, none of these differences were statistically significant using one‐way ANOVA tests. We used multiple linear regression models to determine which variables were associated with higher total quality scores. The variables in the final model that were significantly associated with total report quality were the inclusion of any primary literature citations (*β* = 1.36, *p* < 0.001) and the inclusion of any patient resources (*β* = 1.72, *p* < 0.001). The total variance explained by our final model was ~72% (adjusted *r^2^*).

### 3.5 Direct comparison of reports

Three findings occurred three times each in this series. No statistics were assessed for this subset of data. However, these data serve to illustrate the variability in reporting practices. Reporting characteristics and metrics varied between laboratory type for word count, total citations, citation types, quality scores, and readability (Table 5).

**Table 5:** Comparison of individual reports for recurrent finding

Laboratory information		Descriptive qualities					Quality ratings			Readability
Laboratory	Type	Word count	Total pages	Primary literature cited	Database cited	Total citations	Background	Information	Total	SMOG level
22q11.2 deletion syndrome										
Lab A	Specialty	393	3	Y	Y	10	5.0	5.0	5.0	17.6
Lab J	Reference	159	2	N	Y	1	2.5	1.0	1.9	19.3
Lab T	University	324	2	Y	Y	6	2.2	2.8	2.4	20.2
Wolf–Hirschhorn syndrome										
Lab D	Specialty	275	X	Y	Y	2	3.5	1.3	2.4	19.5
Lab M	Hospital	174	3	N	Y	3	1.2	3.3	2.0	19.9
Lab M	Hospital	103	2	N	Y	3	1.2	3.3	2.0	19.5
15q11.2 BP1‐BP2 deletion										
Lab I	Reference	194	X	Y	Y	4	3.8	4.0	3.9	17.1
Lab O	Hospital	184	X	N	N	0	1.2	1.0	1.1	18.5
Lab V	University	348	X	Y	N	3	5.0	2.0	3.0	17.0

Note

Y: Yes; N: No; X: unable to be assessed due to incomplete report or due to multiple findings in a single report.

For each finding, there was large difference in word count between the longest and shortest report: 234 words (22q11.2 deletion syndrome), 172 words (Wolf–Hirschhorn), and 164 words (15q11.2 BP1‐BP2 deletions). In each of these cases, the reports from HLs were the shortest. There was also a wide range in citation practices, with one HL citing zero references in their report for a 15q11.2 BP1‐BP2 deletion, while others reported 6 (UL) and 10 (SL) for 22q11.2 deletion syndrome. In none of the reports did an HL cite primary literature. Report quality ranged from 1.1 for a report about a 15q11.2 BP1‐BP2 deletion to 5.0 for a report about 22q11.2 deletion syndrome. SMOG scores varied minimally, with the lowest at 17.0 for a report on a 15q11.2 BP1‐BP2 deletion and the highest at 20.2 for a report about 22q11.2 deletion syndrome. This is a total range of 3.2 grade levels.

### 3.6 Correlations among variables

Bivariate analyses were conducted to explore the relationships between the various report characteristics (Supporting information Table S2). As would be expected, word count was significantly correlated with document length (*r* = 0.494, *p* ≤ 0.001), but interestingly it was also associated with the number of primary literature citations (*r* = 0.424, *p* ≤ 0.01) and the DISCERN Background quality measure (*r* = 0.381, *p* ≤ 0.05). Document length was also associated with DISCERN Background and Information quality ratings (*r* = 0.386, *p* ≤ 0.05 and *r* = 0.454, *p* ≤ 0.01), the total number of citations (*r* = 0.564, *p* ≤ 0.001), and the total number of primary literature citations (*r* = 0.397, *p* ≤ 0.01), but not database citations.

## 4 DISCUSSION

We have provided a broad assessment of characteristics, readability, and quality for genomic testing reports that offers a view of the current state of laboratory result communication. We found that over a third (9 laboratories, ~35%) did not include one or more element from the ACMG reporting guidelines. Additionally, few laboratories included non‐ACMG elements, with three laboratories (11.5%) completely omitting these elements and only four laboratories (15.4%) including at least half of these elements. Also notable is the fact that laboratories do not regularly include treatment or management information in reports when that information is relevant and available (often for free). Thus, there is a wide disparity in reporting practices as well as how and what information is reported.

Some of these disparities (as well as document length and word count) may be due to how laboratories interpret CLIA regulations as to what information is required such that results “include pertinent information required for interpretation” (CFR 42 §493.1445 (e)(8)). Alternatively, these disparities may be in part due to factors beyond the control of a laboratory, such as integrating and communicating results from a laboratory directly to an EHR system via a standardized system (e.g., Health Level 7). Lastly, it is possible a laboratory has multiple report versions, one that is formatted for an EHR and another that is sent to providers. Both interpretation of CLIA regulation and EHR integration may impact not only the formatting, but also the type of information that can be presented, such as a table or references section. Ultimately, we cannot rule out that these factors played a role in our results and are unable to fully account for them in these analyses. Regardless, these differences may limit effectively communicating results to the provider and, perhaps, impact downstream medical management.

The only characteristic that differentiated the reports between laboratory types was the use of the Model format style (for an example, see Supporting information Figure S1). Report formatting mostly followed the two most popular styles, the Model format and the “standard” format (i.e., a genetic test result printed on a biochemical test result template). The fact that RLs rarely used the Model format may be due to the fact that testing at a large volume requires a more rigid reporting pipeline with fewer distinct templates and elements. Additionally, RLs and HLs may need to adhere to specific standards (e.g., Health Level 7) when reporting the test results, which may limit their ability to use alternate formats or different reporting elements. Regardless, a survey of satisfaction and perceptions of usability of genetic report formats from almost 400 primary care physicians found that the Model format was universally preferred. It received higher average scores than the “standard” report by providers in all areas assessed and aided in easier report usage including finding the result, interpreting the result, and medical decision making (Scheuner et al., 2013). This is not surprising, as the original template for the Model style was developed using multiple stakeholders, including generalists, genetic specialists, health educators, information technology specialists, health insurance professionals, and consumers in order to facilitate better communication (Scheuner et al., 2012). Thus, the Model format likely communicates genomic results more effectively than other formats.

Perhaps surprisingly, Background quality was significantly correlated with measures of document length, total and primary literature citations, and the number of patient resources. This seems to indicate that longer reports with more primary literature citations tend to offer more in‐depth information regarding the associated condition, genomic finding, and/or risk for a finding to cause or increase susceptibility for symptoms. As primary literature citations, but not database citations, were also correlated with quality of Information Reliability, word count, and document length, it could be concluded that laboratories that routinely cite primary literature have higher quality reports and perhaps inclusion of primary literature sources could be used as a proxy for general quality. The same can be said for patient resources, which are also strongly correlated with both quality scores for Background and Information Reliability.

Despite ACMG published guidelines for information to include in CMA reports, the specific elements included within a given report varied substantially by laboratory with several laboratories not routinely including all elements. Omissions included descriptions of gene content within the specific CNV, a statement of CNV classification (e.g., “pathogenic”), citations from the literature, and follow‐up recommendations. Omitting the classification statement may have real‐world implications, as a provider (or patient/family) may incorrectly interpret the reported CNV for a set of symptoms and seek inappropriate or unnecessary testing or fail to seek additional testing (e.g., sequencing) to determine whether the condition is caused by some other class of genetic abnormality (e.g., a sequencing variant). Additionally, there was one laboratory that described an inherited CNV with confusing and contradictory terminology including both “abnormal” and “unknown significance” in one report's classification statement. These types of errors or omissions could confuse a provider when interpreting a genetic test and may ultimately lead to incorrect follow‐up strategy, wasted time as a provider attempts to clarify the meaning of the result, or legal action against a provider or laboratory. Given the recent (and successful) lawsuits brought by patients against providers and laboratories, this is not a theoretical concern (Marchant & Lindor, 2013; Ray, 2017). Furthermore, these inconsistencies could also cause an erosion of the provider's trust with the laboratory or genetic testing process in general.

Two other ACMG elements that were used inconsistently in this series of reports were including either the genes involved in the genetic alteration or supporting literature. These omissions are likely to have a greater impact for findings classified as having unknown or uncertain significance. This information can be critical for re‐analysis of a genetic finding to determine whether newer research has implicated one or more genes or the type of genetic alteration in a newly described condition. Also, as future human reference genomes are released, the genes within a CNV may change and thus knowing the original location of the finding and the genes involved is helpful when and if re‐analysis of a CNV is needed. As more providers without specialty genetics training order genomic tests, including gene content will be critical as providers may want to search the medical literature to see whether there are updated articles for their patient, yet the provider may not have the technical skill or knowledge to use the specialty genetics databases or genome browsers to find the gene content. Thus, this omission further limits a provider's and patient's or family's ability to find appropriate information and a potential diagnosis.

Non‐ACMG elements were also used inconsistently between laboratories. This lack of inclusion is likely due to numerous factors, including historical practices, expense, reporting workflow, and communication between EHRs or other systems. Including these elements in many cases may help to clearly and effectively communicate the test result, as well as provide information that may impact clinical care. Only one laboratory included management information when possible, though we recognize that omitting guideline‐specific information may be due to historical practices. However, including a section for “Next steps” or “Guidance” for resources or management guidelines for well‐known genetic conditions will help ensure providers are aware of treatment or management options and may improve allocation of clinical resources. Only two laboratories provided patient‐related educational and support information. A curated section for patient‐related information, such as educational or support resources, can be helpful, as there is considerable misinformation on the Internet. While specialty trained professionals, such as genetic counselors and geneticists, can readily identify helpful resources, other providers may not have the resources, other providers may not have the domain‐specific knowledge or time to do this for every patient. Lastly, including a patient‐facing report is not only preferred by families, but also providers (Stuckey et al., 2015; Williams et al., 2016). Strictly defined, this element was not included by any laboratory in this series. This is possibly due to the time‐ and resource‐intensive nature of creating this type of information and integrating it into the reporting pipeline. However, one laboratory (Lab A) did include components that were patient‐facing, such as educational resources, an explanation of genetic counseling, and an initial summary of the finding that is similar to the “Interpretive summary” proposed by Haga et al. (2014).

In terms of readability, SMOG scores were similar between laboratory types, with the median and average SMOG score was equivalent to a masters‐level graduate. This is a higher reading level than other clinical reports. One study reviewed >55,000 radiology reports from multiple specialties and found that the average reading level varied from a high school junior to college sophomore, while a review of 240 neuropsychiatric reports found average reading levels between a high school sophomore and college freshman (Baum et al., 2018; Trofimova, Vey, Safdar, Duszak, & Kadom, 2018). From this limited information, it may be concluded that genomics reports are more difficult to read. Although most providers are capable of reading at the levels in the genomic reports, it may be a burden for many providers who are pressed for time or have less familiarity with genetics jargon. Efforts to lower readability by removing or rewording unnecessarily complex writing (i.e., jargon) may improve provider (and patient) comprehension. However, it has been argued that reducing writing complexity can lead to vagueness, decrease accuracy, and other negative downstream effects when interpreting results (Baum et al., 2018). Thus, a balance between clarity and simplicity is key to improving readability while maintaining quality.

It is also notable that this readability level is too high for patients and their families. The average reading level in the United States is estimated to be around 8th grade (Houts, Doak, Doak, & Loscalzo, 2006). Foe and Larson (2016) recommend a reading level of health information materials to be between the 6th and 8th grade levels. This means the average report from any laboratory is substantially beyond most patient's or family's reading comprehension. Unfortunately, genetic information is often important not only to the patient but also to the extended family. Providing a family section may help not only the patient and provider, but also extended at‐risk family members.

Our quality analysis showed a wide range of quality for all laboratory types. Background quality scores were more strongly correlated with primary literature citations than with database citations in bivariate analysis and statistically significant in multiple linear regressions. This seems to suggest that laboratories that regularly access primary literature are more likely to include detailed information about a condition or finding leading to inclusion of more detailed background information, whereas laboratories that rely on databases, like OMIM, may include only general information. If this is so, it is likely because reference databases condense primary literature in their editorial process, omit certain information, or have out‐of‐date/incomplete information about a gene or condition. While laboratories are likely to cite only databases for multiple reasons, one is likely due to limited time or resources to assess and cite primary literature. Using only databases as a reference likely allows for adequate or high report quality in well‐characterized conditions or genes, but likely lead to less detailed information for variants that are classified as likely pathogenic or of uncertain/unknown significance.

Included in the Background quality score was an assessment of whether a laboratory included information about published management and treatment guidelines or resources. There were eleven reports of conditions that could have included relevant information regarding management or treatment, such as for conditions like Down syndrome and Williams syndrome. In all ten instances in which ULs, HLs, or RLs identified such a condition, the report did not cite these references or discuss them in the text. However, one SL (Lab A) cited published guidelines and/or recommendations in the single report for which relevant guidelines existed (22q11.2 deletion syndrome).

While from a strict regulatory standpoint it is not the duty of a laboratory to report that clinical management guidelines exist, laboratories play a collaborative role in patient care. One example of this is seen when laboratories expedite certain testing due to a patient's worsening symptoms. The ordering provider, who is charged with integrating the results of a report into the clinical care of the patient, may not be aware that guidelines for a specific diagnosis exist, may not know where to find them, or may not have the time to find them. However, providers often do provide follow‐up recommendations for a patient after an unknown/uncertain or pathogenic finding is reported (Hayeems et al., 2015). Since many laboratories conduct a new (or updated) literature review when writing a report, inclusion of this information into their current workflow is feasible. Furthermore, there is evidence that providers who are not formally trained in a specific area of medicine are less likely to provide management consistent with published guidelines (Dhar et al., 2011). Therefore, we believe including language making the provider aware of and a citation to management‐related information could be considered a “best practice,” as this could reduce lag time for referrals and implementation of interventions for the many conditions identified by CMA for which management guidelines exist (Riggs et al., 2014).

Lastly, a comparison of single laboratory reports for the same condition more readily demonstrates the variability between laboratories. All laboratories presumably have access to much of the same primary literature and likely use a template report for these three findings. However, there are clear differences between these reports. Some of these differences may be due to the specific finding, such as a larger, atypical deletion that includes more genes and requires more information (and potentially more citations). But other differences are likely due to laboratory practices, such as whether to cite references at all. These differences are likely to be of interest to some providers and may lead to differences in communication effectiveness. Interestingly, in this subset, hospital reports were the shortest and did not cite primary literature references. Given that primary literature citations (as a categorical variable) and not total citations were highly and positively associated with overall report quality, inclusion of any primary literature may be an easy way to get a general idea of a report's quality. Differences in word count were also considerable between individual reports. It is possible some providers, like geneticists, would prefer a short report without much information on these conditions, as they are likely familiar with the findings, management, and implications for their patient. However, generalists or nongenetics specialists may find a more in‐depth report (i.e., a slightly longer report) to be more helpful when determining potential follow‐up evaluations and referrals.

Based on our analyses, we propose the following recommendations as areas for improvements in reporting of genomic findings:

1. Adhering to the ACMG recommendations. The ACMG has set out minimum standards for reporting findings for CMA. Laboratories should review these basic elements and ensure that their reports meet these basic standards. Including these may help in communicating results and with efforts to reclassify findings of unknown significance.
2. The Model format is preferred and has been shown to be effective (Scheuner et al., 2013). Laboratories should consider adapting this format.
3. Citation of sources is a key element of reporting. Laboratories should consider adding a dedicated section after the interpretation for these references. At minimum, this could include PubMed IDs or URLs to database websites or language identifying the use of proprietary databases. Although this would be less helpful for families, it would give providers or research groups a starting point when trying to understand the information used in the report. A fully formatted citation like those seen in academic articles is likely to be clearest and most useful. For electronic reports, adding article hyperlinks to citations can also increase utility for providers and patients.
4. Citing peer‐reviewed, published information concerning suggested medical management, treatments, or surveillance is a valuable addition to reports. Many laboratories already include a section for “Recommendations,” which is often limited to a general referral to genetic counseling. This section could be more effective with citations for known management guidelines or treatment recommendations. Of note, if laboratories do proceed with this, they could rename this section “Considerations” or “Guidance” (Williams et al., 2016).
5. A “Patient Resources” section can be helpful. There are several well‐known groups to cite, such as Unique (the Rare Chromosome Disorder Support Group), and others listed on well‐known reference websites, like GeneReviews or Genetics Home Reference. A side benefit of adding these resources is that providers may also find this information helpful.
6. To improve readability and utility for patients and families, laboratories should consider reducing the readability level of their provider‐facing report and/or adding readily accessible family‐friendly resources. Removing common genetics jargon and replacing it with simpler language could be considered (e.g., “haploinsufficiency” can become “loss of one gene copy is what causes symptoms”).

We recognize that some of these considerations would result in substantial changes to reporting practices and workflow and may impose significant up‐front and downstream costs. We also recognize that laboratories have different reporting workflows such that specific parts are out of their control, as they may need to adhere to standards for EHR integration or contract out report writing. When and where laboratories do have control over their reports, we encourage laboratories to consider implementing some or all of these points or discussing these points with their partner companies. Importantly, several of these are one‐time changes that could improve laboratory report utility, such as adding a references section. These suggested improvements are also in line with provider preferences, such as using the Model format and providing references to known treatments or management guidelines. Lastly, a report is a technical document and will likely always be written for a healthcare provider and not a family/patient. Thus, while improving reports for families/patients is important, it should not be done to reduce the utility for a provider.

### 4.1 Limitations

There are several limitations to this study. This dataset represents a convenience sample, some laboratories were represented based on a single report, and several reports were incomplete. The small subgroup population limited our ability to find statistically significant differences between laboratory types. Subsequent studies should seek a larger sample size from a more varied group of laboratories. Additionally, we did not analyze reports that used next‐generation sequencing, which may include different characteristics and reporting elements. Furthermore, our quality data are not meant to encapsulate all forms of report quality, so issues such as report errors or typographical errors were not assessed by this score. Our assessment of reporting elements may be inaccurate in some cases, as it is possible that a laboratory provided a patient‐only report and this report was not included in our series. Lastly, the quality data are subjective and one of the two raters works for a specialty laboratory whose reports are included in this series.

### 4.2 Future research

Our analysis excluded comparisons between patient‐facing reports and provider reports. These patient‐facing reports are intended to promote client understanding by defining or removing medical and genetics terminology, thus lowering the reading difficulty. Future analyses should include patient‐facing reports. Additionally, future researchers could focus on which elements aid in effective communication for a provider and family to determine which reporting elements are best to include. Lastly, analysis of other types of genetic testing reports, such as sequencing results, will be helpful to determine whether differences exist between testing methodologies and reporting practices.

## 5 CONCLUSIONS

This retrospective analysis illustrates the diversity of practices in reporting genetic findings and indicates that there is room for improvement in CMA test reports. Our study identified differences in reporting elements for different laboratories, including which reporting elements are included in a report, how the report is formatted, and a laboratory's practice of reporting condition‐related resources or guidelines. Laboratories of all types may consider updating their current reporting practices by considering previously reported provider preferences, which may help effectively communicate findings and lead to follow‐up from CMA tests. This could positively impact the translation of genetic testing into a patient's medical management and personalized care, especially as genetic testing becomes more widely utilized.

## CONFLICTS OF INTEREST

This study was funded by Lineagen, Inc. Three of the authors (Kyle Davis, Megan Martin, and Robert Wassman) are or were employed by Lineagen, Inc., and all have stock options in Lineagen.
