# Supplementary figures and images for: A comparison of genomic laboratory reports and observations that may enhance their clinical utility for providers and patients
Source: Mol Genet Genomic Med. 2019 May 21;7(7):e00551. doi: 10.1002/mgg3.551 (PMC6625363; doi:10.1002/mgg3.551)

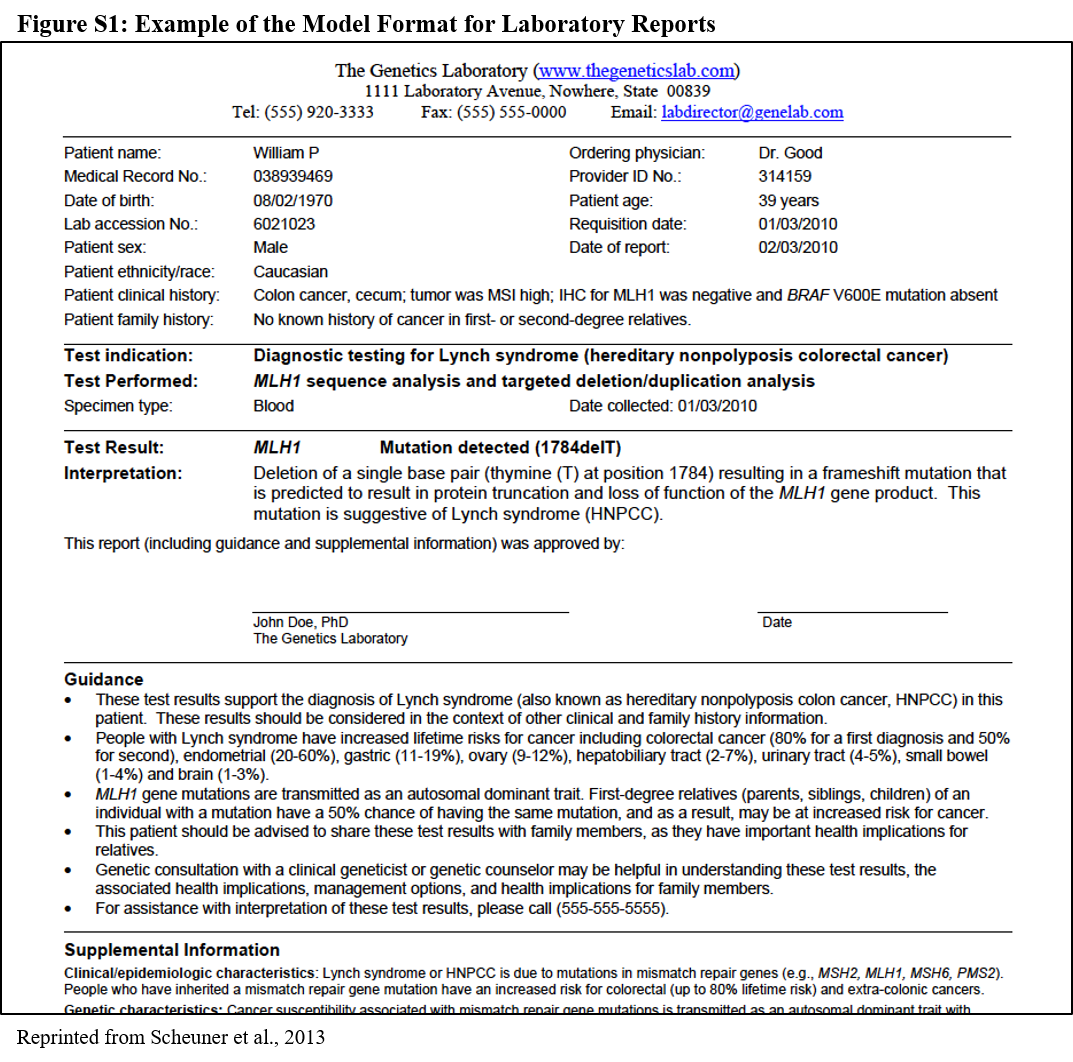

Supplement: Supplementary file 1 [file MGG3-7-e00551-s001.tif]
