# Supplementary material for: A comparison of genomic laboratory reports and observations that may enhance their clinical utility for providers and patients
Source: Mol Genet Genomic Med. 2019 May 21;7(7):e00551. doi: 10.1002/mgg3.551 (PMC6625363; doi:10.1002/mgg3.551)
Supplement: Supplementary file 2 [file MGG3-7-e00551-s002.docx]

**Table V: Cohen's Kappa Statistics for Quality Ratings**

| **Standard Weighting** | | | | |
| --- | --- | --- | --- | --- |
| DISCERN-Genetics Area | Percentage  Agreement | Kappa  statistic | Std.  Error | *Z*-score |
| Background | 59.1% | 0.49*** | 0.07 | 6.5 |
| Management/treatment | 92.3% | 0.64** | 0.20 | 3.2 |
| Risk | 28.0% | 0.14* | 0.08 | 1.7 |
| Support | 63.6% | 0.5*** | 0.09 | 5.8 |
| Resources | 65.9% | 0.55*** | 0.08 | 6.8 |
| *Average* | *61.8%* | *0.46* |  | |
| **Unique Weighting** | | | | |
| Background | 68.2% | 0.49*** | 0.08 | 5.8 |
| Management/treatment | 92.3% | 0.64** | 0.20 | 3.2 |
| Risk | 34.4% | 0.09 | 0.09 | 0.96 |
| Support | 78.2% | 0.64*** | 0.11 | 5.9 |
| Resources | 75.0% | 0.65*** | 0.09 | 7.3 |
| *Average* | *69.6%* | *0.61* |  | |

**p*≤0.05; ***p* ≤0.001; ****p* ≤0.0001
