# Supplementary material for: A comparison of genomic laboratory reports and observations that may enhance their clinical utility for providers and patients
Source: Mol Genet Genomic Med. 2019 May 21;7(7):e00551. doi: 10.1002/mgg3.551 (PMC6625363; doi:10.1002/mgg3.551)
Supplement: Supplementary file 3 [file MGG3-7-e00551-s003.docx]

**Table S2: Bivariate Correlations for Report Characteristics**

|  | Lab Type | 2 | 3 | 4 | 5 | 6 | 7 | 8 | 9 | 10 | 11 | 12 |
| --- | --- | --- | --- | --- | --- | --- | --- | --- | --- | --- | --- | --- |
| 2. Classification | 0.061 |  |  |  |  |  |  |  |  |  |  |  |
| 3. Test Year | - 0.060 | 0.292 |  |  |  |  |  |  |  |  |  |  |
| 4. Word Count | - 0.002 | 0.004 | 0.047 |  |  |  |  |  |  |  |  |  |
| 5. Page Length | - 0.281 | 0.063 | 0.098 | 0.494*** |  |  |  |  |  |  |  |  |
| 6. Model Format | - 0.061 | - 0.273 | - 0.097 | - 0.014 | - 0.146 |  |  |  |  |  |  |  |
| 7. Total Citations | - 0.233 | 0.034 | 0.145 | 0.247 | 0.564*** | - 0.034 |  |  |  |  |  |  |
| 8. Primary Literature Citations | - 0.066 | - 0.047 | - 0.137 | 0.424** | 0.397** | 0.140 | 0.660*** |  |  |  |  |  |
| 9. Database Citations | 0.009 | - 0.144 | - 0.033 | 0.021 | 0.228 | - 0.144 | 0.378* | 0.316* |  |  |  |  |
| 10. Patient Resources | - 0.329* | - 0.098 | 0.218 | 0.212 | 0.276 | 0.098 | 0.604*** | 0.221 | 0.200 |  |  |  |
| 11. Background Quality | - 0.230 | - 0.283 | - 0.043 | 0.381* | 0.386* | 0.229 | 0.552*** | 0.636*** | 0.266 | 0.430** |  |  |
| 12. Information Quality | - 0.173 | - 0.035 | - 0.070 | 0.247 | 0.454** | 0.121 | 0.633*** | 0.653*** | 0.268 | 0.518*** | 0.519*** |  |
| 13. SMOG score | - 0.022 | 0.330* | 0.065 | 0.085 | - 0.080 | - 0.114 | - 0.010 | - 0.179 | - 0.082 | - 0.009 | 0.060 | - 0.081 |

**p*≤0.05; ***p* ≤0.01; ****p* ≤0.001
